# Clinical Features and Genetic Spectrum of Patients With Clinically Suspected Hereditary Progressive Spastic Paraplegia

**DOI:** 10.3389/fneur.2022.872927

**Published:** 2022-04-28

**Authors:** Yuzhi Shi, An Wang, Bin Chen, Xingao Wang, Songtao Niu, Wei Li, Shaowu Li, Zaiqiang Zhang

**Affiliations:** ^1^Department of Neurology, Beijing Tiantan Hospital, Capital Medical University, Beijing, China; ^2^China National Clinical Research Center for Neurological Diseases, Beijing, China; ^3^Monogenic Disease Research Center for Neurological Disorders & Precision Medicine Research Center for Neurological Disorders, Beijing Tiantan Hospital, Capital Medical University, Beijing, China; ^4^Department of Functional Neuroimaging, Beijing Neurosurgical Institute, Beijing, China

**Keywords:** hereditary spastic paraplegia, spinocerebellar ataxia, next generation sequencing, dynamic mutation, triplet repeat primed PCR

## Abstract

**Background and Purpose:**

A variety of hereditary diseases overlap with neurological phenotypes or even share genes with hereditary spastic paraplegia (HSP). The aim of this study was to determine the clinical features and genetic spectrum of patients with clinically suspected HSPs.

**Methods:**

A total of 52 patients with clinically suspected HSPs were enrolled in this study. All the patients underwent next-generation sequencing (NGS) and triplet repeat primed PCR to screen for the dynamic mutations typical of spinocerebellar ataxia (SCA). Multiplex ligation-dependent probe amplification (MLPA) was further conducted in patients with no causative genetic mutations detected to examine for large deletions and duplications in genes of *SPAST, ATL1, REEP1, PGN, and SPG11*. Clinical characteristics and findings of brain MRI were analyzed in patients with definite diagnoses.

**Results:**

The mean age of the patients studied was 36.90 ± 14.57 years. 75% (39/52) of patients manifested a phenotype of complex form of HSPs. A genetic diagnosis was made in 51.9% (27/52) of patients, of whom 40.3% (21/52) of patients had mutations in HSPs genes (*SPG4/SPG6/SPG8/SPG11/SPG15/SPG78/SPG5A*) and 11.5% (6/52) of patients had mutations in SCAs genes (*SCA3/SCA17/SCA28*). SPG4 and SPG11 were the most common cause of pure form of HSPs (5/6, 83.3%) and complex form of HSPs (5/15, 33.3%), respectively. Gait disturbance was the most common initial symptom in both the patients with HSPs (15/21) and in patients with SCAs (5/6). Dysarthria and cerebellar ataxia were detected in 28.5% (6/21) and 23.8% (5/21) of patients with HSPs, respectively, and were the most common symptoms in addition to progressive weakness and spasticity of the lower limbs. Cerebellar atrophy was seen on the brain MRI of patients with SPG5A, SCA3, and SCA28.

**Conclusion:**

Causative genetic mutations were identified in 51.9% of patients with clinically suspected HSPs by NGS and triplet repeat primed PCR. A final diagnosis of HSPs or SCAs was made in 40.3% and 11.5% of patients, respectively. The clinical manifestations and neuroimaging findings overlapped between patients with HSPs and patients with SCAs. Dynamic mutations should be screened in patients with clinically suspected HSPs, especially in those with phenotypes of complex form of HSPs.

## Introduction

Hereditary spastic paraplegia (HSP) is a heterogeneous group of neurodegenerative disorders characterized by progressive weakness and spasticity of the lower limbs resulting from pyramidal tract dysfunction (1). HSP can be classified as either pure or complex forms according to the clinical symptoms and signs (1). Pure forms involve lower limb spastic paraplegia and may include bladder disturbances and subtle sensory signs such as impaired vibration sense. Complex forms include additional neurological and nonneurological manifestations such as cognitive impairment, dysarthria, optic atrophy, peripheral neuropathy, and seizures.

The modes of inheritance of HSPs include autosomal dominant (AD), autosomal recessive (AR), X-linked, mitochondrial, or maternal modes of inheritance (2, 3). The Online Mendelian Inheritance in Man (OMIM) has listed more than 80 distinct genetic forms of HSPs to date. With the introduction of next-generation sequencing (NGS), several different approaches to genetic testing for HSPs can be adopted: targeted sequencing gene panels, whole-exome sequencing (WES), and whole-genome sequencing (WGS). NGS-based gene panels for specific conditions or groups of conditions are cost-efficient and widely available. Unlike targeted gene panels, WES has the strength to detect exon mutations with a hypothesis-free approach compared with the targeted gene panels by allowing for the potential interrogation of many relevant genes. However, both the targeted sequencing gene panels and WES have limitations, for example, they will generally not identify structural variants (SVs) or deep intronic variants (4). These limitations can be resolved by WGS, which has the capacity to identify large deletions/duplications, as well as promoter, deep intronic, and mitochondrial genome mutations. However, the high expense and difficulties of processing, storing, and interpreting the large amounts of genomic data of WGS limit its clinical application. For cost efficiency, targeted sequencing gene panels and WES are the common choices for clinicians to detect the genetic causes to patients clinically suspected HSPs. Currently, WES has become the preferred choice in the recent years due to the large decrease in the cost and the width of mutation detection. Although the identification of copy number variants (CNVs) from WES or targeted sequencing gene panels data based on read depth information has been proven to be reliable (5, 6), multiple ligation-dependent probe amplification (MLPA) is usually conducted to prove that no existing CNVs are undetected.

A variety of hereditary diseases overlap the neurological phenotypes or even share genes with HSPs such as hereditary cerebellar ataxia, spastic ataxia, inherited neuropathies, leukodystrophies, hereditary amyotrophic lateral sclerosis, monogenic Parkinson's disease, and some hereditary metabolic disorders (7, 8). The majority of these diseases can be differentiated from HSPs by clinical presentations, MRI of the brain and/or spinal cord, or laboratory tests of blood, urine, and/or cerebrospinal fluid (8, 9); others should be differentiated and diagnosed by genetic testing. Genetic causes of the hereditary diseases with slowly progressive spastic paraplegia as the prominent sign are mostly single-gene mutations and that can be detected by targeted gene sequencing or WES. However, some subtypes of hereditary cerebellar ataxia and spastic ataxia, such as spinocerebellar ataxia (SCA) type 1 (*ATXN1*) (10) and type 3 (*ATXN3*) (11) and Friedreich's ataxia (*FXN*) (12), have been reported to present as isolated spastic paraplegia and are caused by abnormal trinucleotide repeat (TNR) expansion. Although WGS has been certified to be reliable for detecting repeat expansion with high sensitivity and specificity (13), targeted gene sequencing or WES is incapable of detecting repeat expansion in routine. Triplet repeat primed PCR (TP PCR) is the usual chosen approach to screen for the abnormal TNR expansion and other dynamic mutations of known SCA genes.

There are some diagnostic strategies based on the clinical-genetic correlations. The genetic diagnosis of HSPs is still a major challenge for clinicians. In this study, we adopted a genetic diagnostic strategy, NGS combined with TP PCR for SCAs, to detect the causative genetic mutation in patients clinically suspected of having HSPs. The aim of this study was to study the clinical features and genetic spectrum detected by the genetic diagnostic strategy described above in patients with clinically suspected HSPs.

## Materials and Methods

### Study Subjects

Fifty-two unrelated patients with clinically suspected HSPs were consecutively enrolled from the outpatient clinics and inpatient Department of Neurology in the Beijing Tiantan Hospital, Capital Medical University, between 2016 and 2019. Patients with slowly progressive spastic paraplegia and fulfilling any one of the following criteria (14) were included: (1) pure spastic paraplegia, (2) spastic tetraparesis with earlier and more severe affection of the lower limbs, and (3) spastic paraplegia as a prominent sign of a degenerative disease affecting several parts of the nervous system. Patients with other causes of the presenting symptoms were excluded from this study (3, 8). MRI of the brain and spinal cord, biochemical tests, tests of deficiency of lysosomal enzymes (arylsulfatase A, β-galactosidase, β-hexosaminidase A/B, β-galactocerebrosidase, and β-glucocerebrosidase) and elevated levels of very long-chain fatty acids, cerebrospinal fluid analyses, and examinations of vitamin B12 and folic acid were conducted to exclude other causes of spastic paraplegia.

The mode of inheritance in patients with a family history was classified as dominant when the symptoms of HSPs were reported in more than one generation and as recessive when affected family members were present in only one generation (15). The patients without a family history were classified as sporadic cases. The disease severity was evaluated by the Spastic Paraplegia Rating Scale (SPRS) (14).

The diagnostic process of all the patients enrolled in this study was determined by at least two senior neurologists and the final diagnoses were made according to variants detected by mutation analyses and clinical details. The MRI of the brain and spinal cord of patients enrolled in this study were interpreted by one senior neuroimaging specialist.

This study was approved by the Ethics Committees of Beijing Tiantan Hospital and written informed consents were obtained from all the patients.

### Mutation Analysis

Genomic DNA was extracted from peripheral blood samples of each patient with the CWE9600 Automated Nucleic Acid Extraction System using the CWE2100 Blood DNA Kit V2 (CWBiotech, China, CW2553).

**Figure 1:**
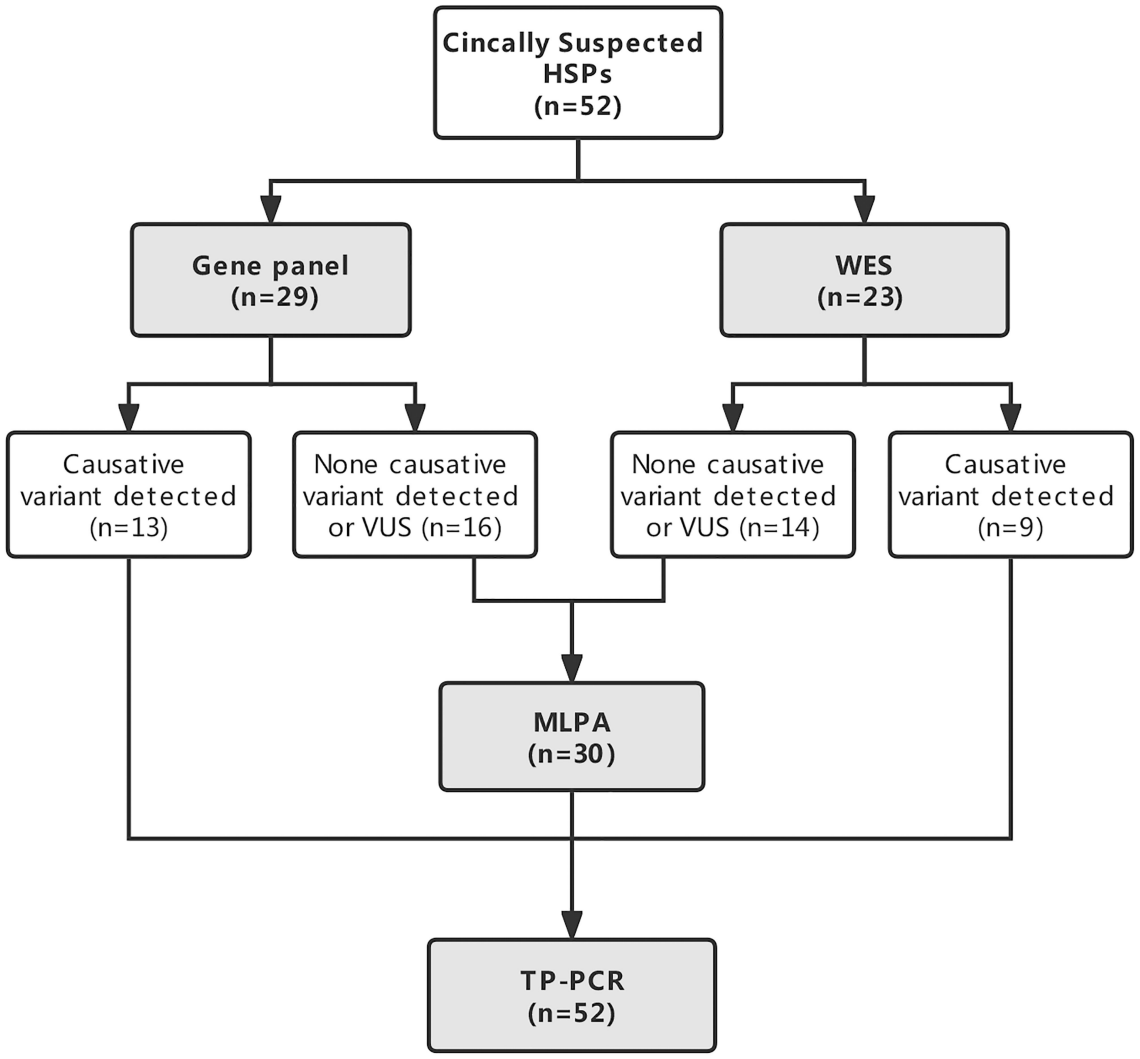
Flowchart of the genetic diagnostic strategy in patients with clinically suspected hereditary spastic paraplegias (HSPs).

All the patients underwent NGS either a targeted gene panel related to the phenotype of progressive spastic paraplegia (*n* = 29) or WES (*n* = 23) and TP PCR to screen for the known dynamic mutations of SCAs. MLPA was conducted in patients without causative variants detected by NGS to further examine large deletions and duplications of some genes related to HSPs. The genetic testing strategy is given in Figure 1.

#### Next-Generation Sequencing

Genomic DNA (gDNA; 750 ng) was fragmented into 200–300 bp lengths by the Scientz08-III Ultrasonic Homogenizer (Scientz, China). The DNA fragments were then processed by end-repairing, A-tailing, and adaptor ligation using the KAPA Library Preparation Kit (Illumina, KR0453, v3.13), followed by an 8-cycle precapture PCR amplification. Then, the amplified DNA sample was captured by a customized panel (Integrated DNA Technologies, USA) related to the phenotype of progressive spastic paraplegia (Supplementary Table 1), IDT xGen Exome Research Panel v1 (Integrated DNA Technologies, USA), and SureSelect Human All Exon v6 (Agilent Technologies, Santa Clara, CA, USA). Captured DNA fragments were purified with Dynabeads MyOne Streptavidin T1 (Invitrogen, Thermo Fisher Scientific, USA) and amplified by 13 cycles of postcapture PCR. The final products were purified by the Agencourt AMPure XP (Beckman Coulter Incorporation, USA) and quantitated with the Life Invitrogen Qubit 3.0 machine and the Qubit dsDNA HS Assay Kit (Invitrogen, Thermo Fisher Scientific, USA). Eventually, quantified DNA was sequenced with 150-bp paired-end reads on an Illumina NovaSeq 6000 platform (Illumina Incorporation, USA) according to the standard manual.

The raw data produced on the NovaSeq platform were filtered and aligned against the human reference genome (hg19) using the BWA Aligner (http://biobwa.sourceforge.net/) after evaluation according to Illumina Sequence Control Software. Single-nucleotide polymorphisms (SNPs) were called by using the Genome Analysis ToolKit (GATK) software (www.broadinstitute.org/gatk). Variants were annotated using ANNOVAR (annovar.openbioinformatics.org/en/latest/). The effects of single-nucleotide variants (SNVs) were predicted by the Sorting Intolerant from Tolerant (SIFT), PolyPhen-2, and MutationTaster programs.

All the variants were interpreted according to the American College of Medical Genetics and Genomics (ACMG) standards and categorized as pathogenic, likely pathogenic, variants of unknown clinical significance, likely benign, and benign. The variants categorized as pathogenic or likely pathogenic were considered to be possible causative mutations and were considered in the final diagnoses by senior neurologists.

#### Sanger Sequencing

Sanger sequencing was carried out to validate the potential variants identified by the targeted gene panel or WES. All the available family members with or without symptoms and signs of HSPs were screened for the confirmed variants for co-segregation analysis.

#### Multiplex Ligation-Dependent Probe Amplification

Patients who had no causative mutation detected by targeted gene sequencing or WES were further examined for large deletions and duplications in SPAST, ATL1, REEP1, PGN, and SPG11 through MLPA analysis using commercially available MLPA kits (SALSA P165-C2; SALSA P213-B2; SALSA P306-B1; and MRC-Holland, Netherlands) according to the manufacturer's recommendations. Quantitative PCR was performed on patient gDNA to investigate the deletions/duplications (primer sequences are available upon request). Intervening sequences were covered with a spacing of ~100 bp. To detect deletion/duplication boundaries, long-fragment PCR was performed for the suggested aberrations following Sanger sequencing.

#### Triplet Repeat Primed PCR

Triplet repeat primed PCR was performed to screen the dynamic mutations of all the known SCA genes, followed by capillary electrophoresis. The primer sequences of all the SCA genes were designed using reference sequences from GenBank and are given in Supplementary Table 2.

The TP PCR assay was carried out with a total volume of 25 μl consisting of the 2× Phanta Max Buffer, 0.4 mM deoxyadenosine triphosphate (dATP), 0.4 mM deoxythymidine triphosphate (dTTP), 0.4 mM deoxycytidine triphosphate (dCTP), 0.4 mM deoxyguanosine triphosphate (dGTP), 0.5 units of the Phanta Max Super-Fidelity DNA Polymerase (Vazyme Biotech Corporation Ltd., China), 0.4 μM of each primer set, and 5× enhancer containing 2.7 M betaine, 6.7 mM DTT, 6.7% dimethyl sulfoxide (DMSO), and 55 μg/ml bovine serum albumin (BSA). Genes were then amplified with the following program: 25 cycles of denaturation at 95°C for 30 s, annealing at 65°C for 30 s, and extension at 72°C for 30 s; then, the reaction temperature was decreased 0.6°C every cycle for another 20 cycles of denaturation at 95°C for 30 s, annealing at 50°C for 30 s, and extension at 72°C for 1 min. The Life ECO Thermal Cycler TC-96/G/H(b)C (Bioer Technology Corporation Ltd., China) was used. PCR products (0.5 μl) were separated by capillary electrophoresis in the ABI3730xl instrument (Applied Biosystems, Foster, CA, USA). The reaction mixture also contained 1 μl of the GeneScan 500 LIZ and 8.5 μl of Hi-Di formamide (Applied Biosystems). Data were analyzed with GeneMapper v4.0 (Applied Biosystems).

### Statistical Analysis

Quantitative features are reported as the mean and SD for normally distributed data. Categorical variables are described by number and percentage. Differences in clinical characteristics between patients with and without definite genetic causes were investigated using the independent *t*-test, the Mann–Whitney *U* test, and the chi-squared test.

IBM SPSS statistics v23.0 software was used for statistical analysis. *P* < 0.05 was considered significant for all the analyses.

## Results

A total of 52 patients with clinically suspected HSPs were enrolled in this study, 38 (73.1%) of whom were male. The mean age of all the patients at examination and onset was 36.90 ± 14.57 and 28.31 ± 15.92 years old, respectively. A family history was reported by 16 (30.8%) patients. The family history suggested a dominant inheritance in 13 (25.0%) patients and a recessive pattern in 3 (5.8%) patients. According to the clinical manifestations, 13 (25.0%) and 39 (75.0%) patients were categorized as having pure form and complex form phenotypes, respectively.

### Genetic Findings

All the patients had their DNA sequenced either on a targeted gene panel related to the phenotype of progressive spastic paraplegia (*n* = 29, 55.8%) or by WES (*n* = 23, 44.2%). Causative variants were detected in 22/52 (42.3%) patients by NGS. Variants of unknown significance were not included. Causative genetic mutations were detected in 44.8% (13/29) of patients sequenced by targeted gene panel and 39.1% (9/23) of patients sequenced by WES among them (*P* = 0.680; Figure 1). No large deletions or duplications of *SPAST, ATL1, REEP1, PGN*, or *SPG11* by subsequent MLPA analysis were found in 30 patients who had no definite causative variants detected by NGS. Abnormal TNR expansion was found in 6 patients by TP PCR. In total, causative genetic mutations related to HSPs and SCAs were identified in 27/52 (51.9%) patients with clinically suspected HSPs by the genetic testing strategy (Table 1). The proportion of causative genetic mutations detected in patients with phenotype of complex form of HSPs (21/39) and in patients with phenotype of pure form of HSPs (6/13) was not significantly different (53.8% vs. 46.2%, *P* = 0.631).

**Table 1:** Genetic causes of the solved cases with clinically suspected hereditary spastic paraplegia.

**Diseases**	**Gene**	**Type of mutation**	**Site**	**Inheritance pattern**	**ACMG**	***N***
**Hereditary spastic paraplegia**						**21**
**Pure form**						**6**
SPG4	SPAST	Nonsense	c.463G>T p.E155X	AD, Het	LP	5
		Missense	c.1413 + 5G>C splicing	AD, Het	LP	
		Frameshift	c.1348_1352del p.R450fs	AD, Het	P	
		Missense	c.1413 + 1G>A splice-5	AD, Het	P	
		Nonsense	c.139A>T p.K47X	AD, Het	P	
SPG78	ATP13A2	Missense & Nonsense	c.1448T>G p.L483R c.92C>G p.S31X	AR, Het	LP	1
**Complex form**						**15**
SPG4	SPAST	Missense	c.1321G>A p.D441N	AD, Het	LP	3
		Missense	c.1139T>G p.L380R	AD, Het	LP	
		Frameshift	c.1212_1216del p.F404fs	AD, Het	P	
SPG6	NIPA1	Missense	c.316G>A p.G106R	AD, Het	P	1
SPG8	WASHC5	Missense	c.1725T>A p.N575K	AD, Het	LP	1
SPG11	SPG11	Frameshift	c.4307_4308delAA p.Q1436Rfs*7	AR, Hom	P	5
		Frameshift	c.733_734del p.M245fs	AR, Hom	P	
		Nonsense & Missense	c.5934_5935insTAACCT GGAA p.V1979_L1980delinsX c.870-2A>G splicing	AR, Het	LP	
		Frameshift	c.6739_6742del p.E2247fs	AR, Hom	P	
		Nonsense & Nonsense	c.5137C>T p.Q1713X c.1435C>T p.Q479X	AR, Het	LP	
SPG15	ZFYVE26	Missense & Nonsense	c.6588 + 1G>A splicing c.6498C>A p.Y2166X	AR, Het	LP	2
		Nonsense & Nonsense	c.4804C>T p.R1602X c.4278G>A p.W1426X	AR, Het	LP	
SPG78	ATP13A2	Nonsense	c.1444C>T p.R482X	AR, Hom	LP	2
		Frameshift	c.1438_1439delTG p.C480Hfs*40	AR, Hom	LP	
SPG5A	CYP7B1	Nonsense	c.334C>T p.R112X	AR, Hom	P	1
**Spinocerebellar ataxia**						**6**
**Complex form**						6
SCA3	ATXN3	Translated CAG repeat	65/59/76/73 repeats	AD	–	4
SCA17	TBP	Translated CAG repeat	42 repeats	AD	–	1
SCA28	AFG3L2	Missense	c.1996A>G p.M666V	AD, Het	P	1

*SCA, spinocerebellar ataxia; SPG, spinal paraplegia; ACMG, American College of Medical Genetics and Genomics; P, Pathogenic; LP, Likely Pathogenic*.

**Figure 2:**
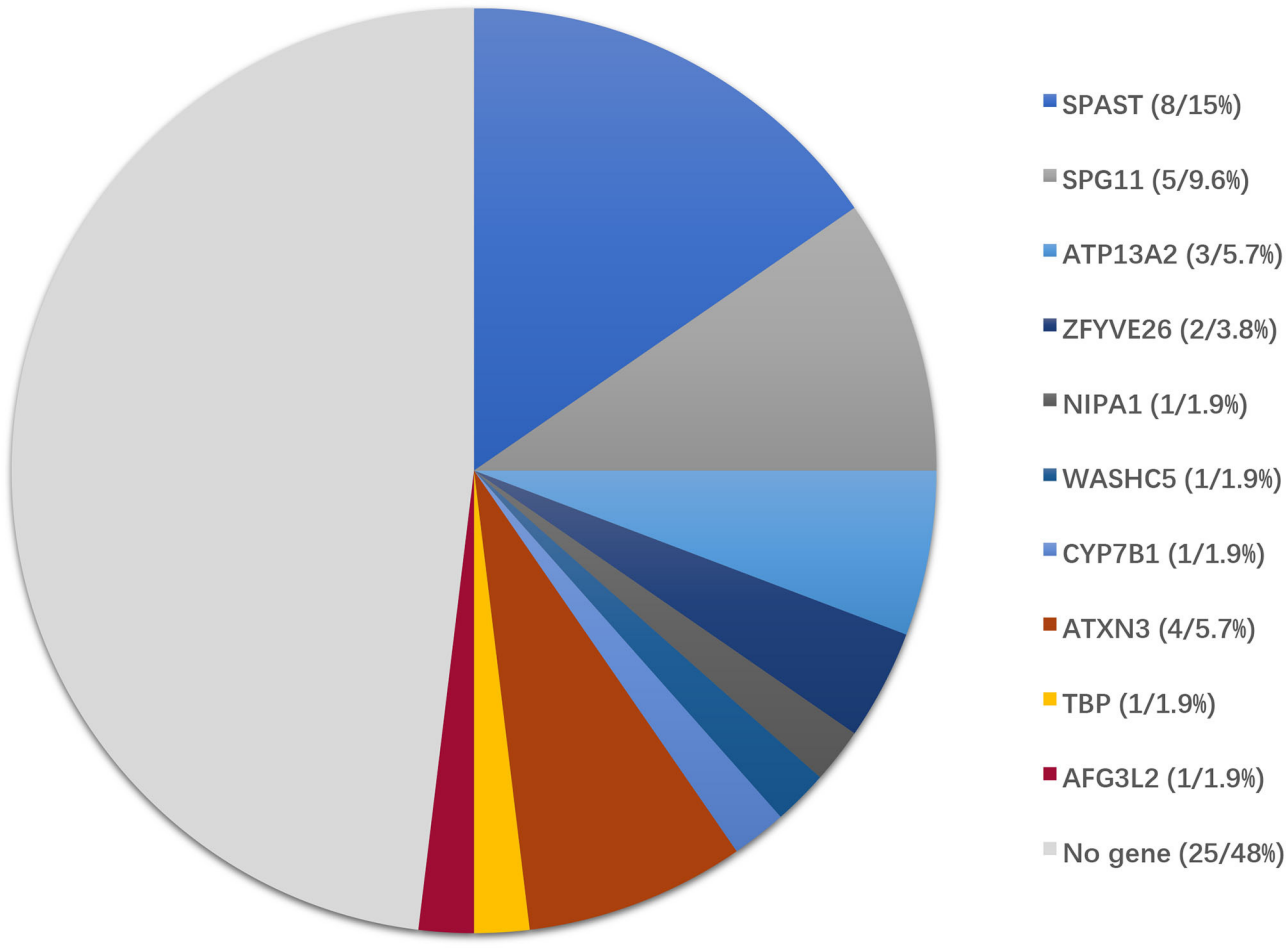
Genotype distribution of patients enrolled in this study. Of 52 patients with clinically suspected HSPs, the final diagnoses were genetically confirmed in 27 patients by the genetic diagnostic strategy.

The causative genetic mutations related to HSPs were identified in 21/52 (40.3%) patients by gene sequencing. Missense, nonsense, or frameshift mutations in *SPAST/SPG4* were identified in 15% (8/52) of patients and were the most common cause of the pure form of HSPs (5/6, 83.3%). Homozygous or compound heterozygous mutations in *SPG11* were detected in 5/52 (9.6%) patients. The vast majority of *SPG11* mutations were frameshift or nonsense mutations. *SPG11* was followed by *ATP13A2/*SPG78 (3/52), *ZFYVE26/*SPG15 (2/52), *NIPA1*/SPG6 (1/52), *WASHC5/*SPG8 (1/52), and *CYP7B1/*SPG5A (1/52) in terms of the frequency of mutation (Table 1, Figure 2).

In addition to the genetic mutations associated with HSPs described above, a variant in *AFG3L2* associated with SCA28 was also identified by NGS. Abnormal CAG repeat expansion in the genes of *ATXN3/*SCA3 (4/52) and *TBP*/SCA17 (1/52) genes was identified by TP PCR (Supplementary Figure 1). Concurrent pathogenic or likely pathogenic genetic mutations were not detected in any patient enrolled in this study (Table 1).

The final diagnosis was made according to clinical information and the results of the genetic diagnostic strategy. Ultimately, the diagnosis of HSPs and SCAs was made in 77.7% (21/27) and 22.2% (6/27) of patients with definite causative genetic mutations, respectively.

### Clinical Features

The clinical characteristics of patients with identified and unidentified genetic causes were compared. No significant difference was found in sex, age, disease duration, family history, initial symptoms, the SPRS score, or genetic sequencing tools adopted between the groups of patients with and without definite genetic causes. The proportion of patients who presented cerebellar ataxia in the group with unidentified causes was much higher than that in the group with identified causes (36.0% vs. 11.1%, *P* = 0.033) and an emotional disorder or psychosis was reported only in the group with identified causes (Table 2).

**Table 2:** Characteristics of the patients with clinically suspected hereditary spastic paraplegia.

**Characteristic**	**Total (n=52)**	**Patients with definite genetic causes (n=25)**	**Patients without definite genetic causes (*n*=27)**	***P***
Male, *n* (%)	38 (73.1)	18 (66.7)	20 (80.0)	0.279
Age at examination (years), mean ± SD	36.90 ± 14.57	35.04 ± 14.41	38.92 ± 14.77	0.342
Age at onset (years), mean ± SD	28.31 ± 15.92	25.48 ± 16.17	31.36 ± 15.37	0.186
Disease duration (years), mean ± SD	8.59 ± 7.52	9.55 ± 7.67	7.56 ± 7.36	0.344
Family history, *n* (%)	16 (30.8)	11 (40.7)	5 (20.0)	0.105
**Clinical features, *n* (%)**				
Weakness of lower limb	48 (92.3)	24 (88.9)	24 (96.0)	0.325
Gait disturbance	51 (98.1)	26 (96.3)	25 (100)	0.249
Weakness of upper limb	11 (21.2)	7 (25.9)	4 (16.0)	0.381
Dysarthria	14 (26.9)	9 (33.3)	5 (20.0)	0.279
Bulbar paralysis	7 (13.5)	5 (18.5)	2 (8.0)	0.259
Bladder disturbances	9 (17.3)	6 (22.2)	3 (12.0)	0.326
Epilepsy	4 (7.7)	2 (7.4)	2 (8.0)	0.936
Cerebellar ataxia	12 (23.1)	3 (11.1)	9 (36.0)	0.033
Extrapyramidal involvement	4 (7.7)	3 (11.1)	1 (4.0)	0.325
Peripheral nerve involvement	6 (11.5)	3 (11.1)	3 (12.0)	0.920
Muscular atrophy	7 (13.5)	5(18.5)	2 (8.0)	0.259
Visual disturbance	6 (11.5)	3 (11.1)	3 (12.0)	0.920
Auditory disturbance	5 (9.6)	1 (3.7)	4 (16.0)	0.123
Skeletal abnormalities	5 (9.6)	3 (11.1)	2 (8.0)	0.703
Cognitive impairment	8 (15.4)	6 (22.2)	2 (8.0)	0.147
Emotional disorder or psychosis	4 (7.7)	4 (14.8)	0 (0)	0.018
Physical retardation	8 (15.4)	5 (18.5)	3 (12.0)	0.438
Intellectual retardation	4 (7.7)	3 (11.1)	1 (4.0)	0.325
SPRS score, mean ±SD	22.1 ± 4.6	23.4 ± 5.7	20.8 ± 7.2	0.794
**Categories according to clinical symptoms and signs**				0.631
Pure form	13 (25.0)	6 (22.2)	7 (28.0)	
Complex form	39 (75.0)	21 (77.8)	18 (72.0)	
**Initial symptom**				0.217
Gait disturbance	35 (67.3)	20 (74.1)	15 (60.0)	
Weakness of lower limb	6 (22.2)	10 (40.0)	16 (30.8)	
Dysarthria	1 (1.9)	1 (3.7)	0 (0)	
**Gene sequencing**				0.278
Targeted gene panel	29 (55.8)	17 (63.0)	12 (48.0)	
WES	23 (44.2)	10 (37.0)	13 (52.0)	

*SPRS, Spastic Paraplegia Rating Scale*.

**Figure 3:**
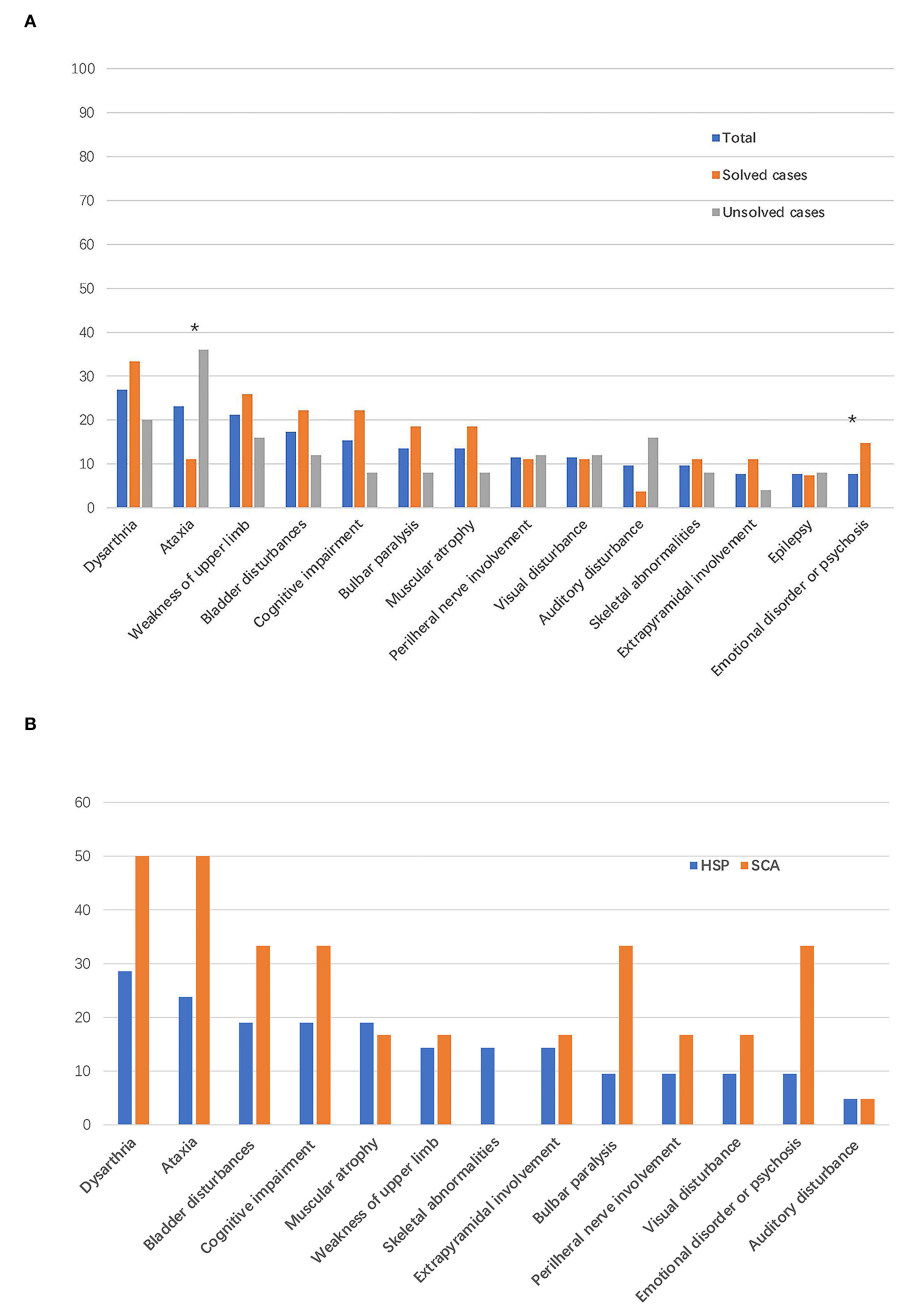
Frequency of complicating signs and symptoms. The bar indicates the proportion of patients with a given sign or symptom. **(A)** Frequency of complicating signs and symptoms in all the patients enrolled in this study and in patients with vs. without defined genetic causes. *P < 0.05. **(B)** Frequency of complicating signs and symptoms in patients with final diagnoses of HSPs and spinocerebellar ataxias (SCAs).

Dysarthria (14/52, 26.9%) was the most common symptom in patients with suspected HSPs, followed by cerebellar ataxia (12/52, 23.1%), weakness of the upper limbs (11/52, 21.2%), bladder disturbances (9/52, 17.3%), and cognitive impairment (8/52, 15.4%; Table 2, Figure 3A), in addition to the dominant symptoms of progressive weakness and spasticity of the lower limbs.

Among the 21 patients with a final diagnosis of HSPs, 6 (28.5%) patients had phenotype of pure form HSPs. In the six patients with pure form of HSPs, five patients were diagnosed with SPG4 and one patient was diagnosed with SPG78 according to the genetic mutations detected. All the patients with final diagnosis of SCAs had complex form of phenotypes. In addition to progressive weakness and spasticity of the lower limbs, the most common symptoms were dysarthria (6/21, 28.5%) and cerebellar ataxia (5/21, 23.8%) in patients with final diagnoses of HSPs. Bladder symptoms (4/21, 19.0%), cognitive impairment (4/21, 19.0%), and muscular atrophy (4/21, 19.0%) were the third most common symptoms in patients with HSPs (Figure 3B). Dysarthria (3/6, 50%) and cerebellar ataxia (3/6, 50%) were the most reported symptoms and signs in patients with a final diagnoses of SCAs (Figure 3B).

Gait disturbance and weakness of the lower limbs as initial symptoms were reported in 67.3% (35/52) and 22.2% (6/52) of patients enrolled in this study, respectively. In patients with HSPs, gait disturbance, weakness of the lower limbs, and dysarthria were reported as the initial symptoms in 15/21, 5/21, and 1/21 patients, respectively. In patients diagnosed with SCAs, gait disturbance and weakness of the lower limbs were reported as the initial symptoms in 5/6 and 1/6 patients, respectively.

### Magnetic Resonance Imaging

**Figure 4:**
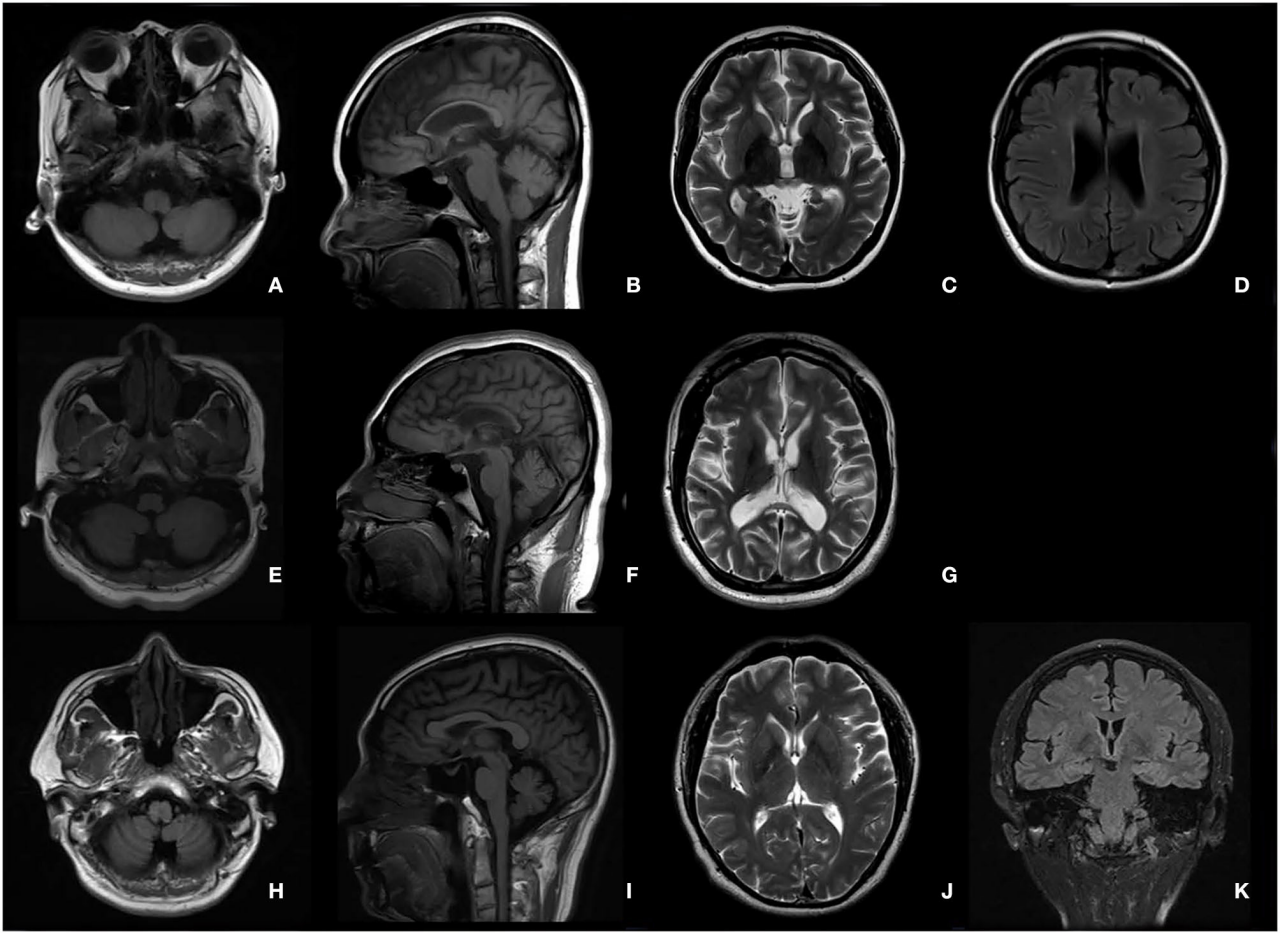
Brain MRI in patients with SPG11 **(A–D)**, SPG15 **(E–G)**, and SPG 5A **(H–K)**. A thin corpus callosum **(B,F)**, “ear of the lynx” sign **(C,G)**, and ventricular dilation **(D,G)** were found in a female patient with SPG11 aged 25 years and a male patient with SPG15 aged 24 years. Periventricular white matter hyperintensities **(D)** are shown in the female patient with SPG11. No cerebellar atrophy was found in the patient with SPG11 **(A)** or in the patient with SPG15 **(E)**. Cerebellar atrophy was the major MRI in the male patient with SPG5A aged 21 years **(H,I)**.

**Figure 5:**
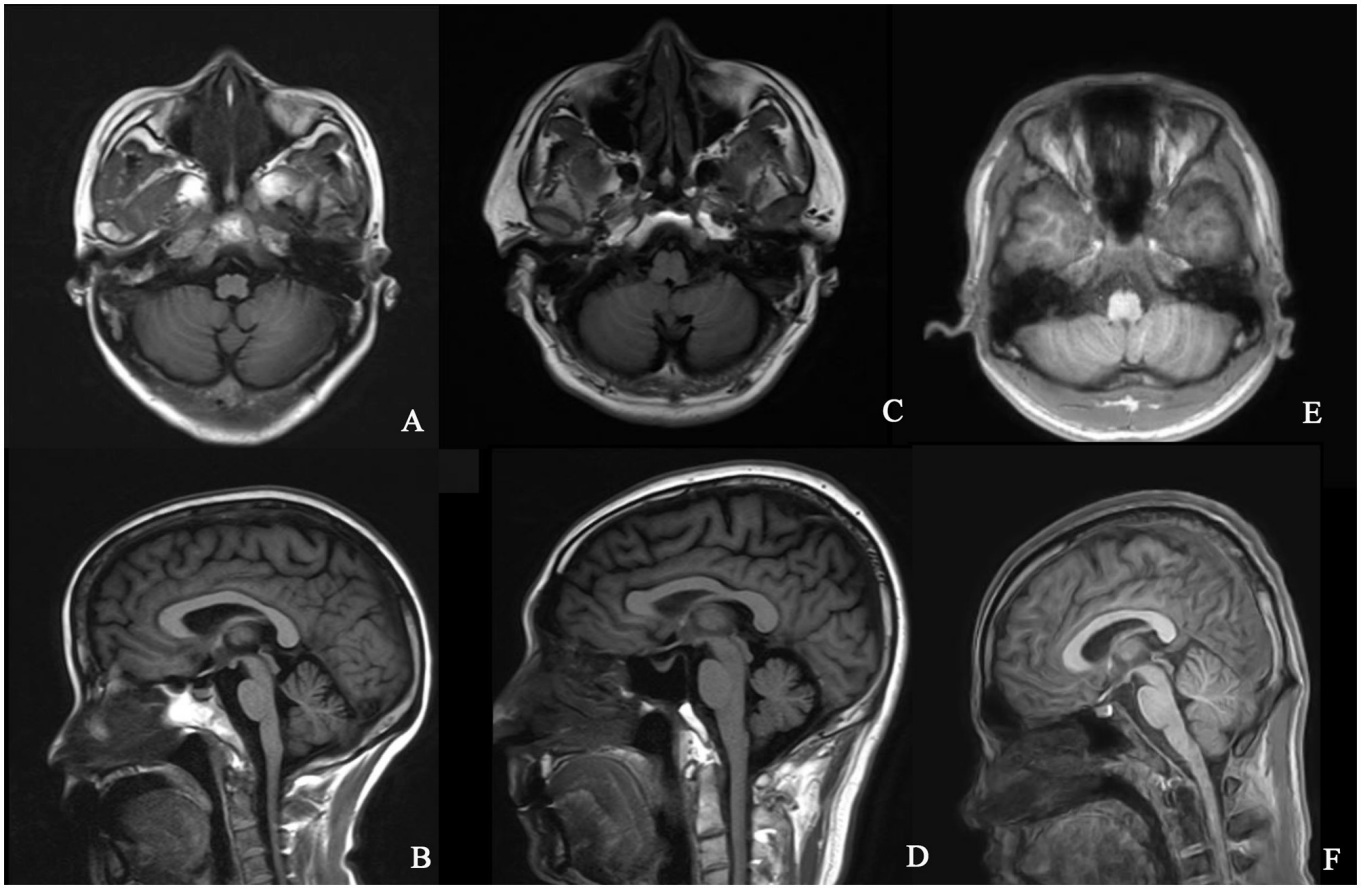
Brain MRI in patients with SCA3, SCA17, and SCA28. T1 axial and sagittal images showing cerebellar atrophy in a 40-year-old female patient with SCA3 **(A,B)**. T1 axial and sagittal images showing cerebellar atrophy in a 50-year-old male patient with SCA28 **(C,D)**. No specific sign of cerebellar atrophy was found in T1 axial and sagittal images of a 17-year-old male patient with SCA17 **(E,F)**.

The typical “ear of the lynx” sign, thin corpus callosum, and ventricular dilatation were seen in all the cases of SPG11 and SPG15 (Figures 4A–G). Periventricular white matter hyperintensities were other MRI findings in 3/5 patients with SPG11 (Figure 4D). In 21-year-old male patient with SPG5A (Figures 4H–K), cerebellar atrophy was detectable on MRI (Figures 4H,I). No significant structural abnormality was found in brain MRI of patients with other genetic causes of HSPs. Cerebellar atrophy was the main MRI finding in patients with SCA3 (Figures 5A,B) and SCA78 (Figures 5C,D). The patient with SCA17 (Figures 5E,F) had a normal cerebellar structure.

## Discussion

In this study, we described the results of genetic analyses of 52 probands with clinically suspected HSPs using a genetic diagnostic strategy based on either a customized panel related to the phenotype of progressive spastic paraplegia or WES combined with TP PCR. We identified causative genetic mutations in 51.9% (27/52) of the patients studied. Despite stringent inclusion and exclusion criteria, we still found one patient with a mutation in the *SCA28* gene by NGS and another five patients with abnormal TNR expansions in *SCAs* genes detected by TP PCR. Final diagnoses of HSPs and SCAs were made in 77.7% (21/27) and 22.2% (6/27) of patients with definite causative genetic mutations, respectively.

In this study, the proportion of patients with sporadic HSPs without a family history was 69.2%, which was much higher than that reported in previous studies (13–47%) (8, 15). The medical referral system is responsible for the gap in the proportions mentioned above to some extent. Patients with a family history, as well as pure form of HSPs, were easier to be recognized and most of them were diagnosed by local specialists of neurology without transfer to our hospital as the national center of neurological diseases for consultation. The higher proportion of patients with complex form of HSPs could also be explained by the same reason (75% vs. 46–49%) (15, 16). In patients with a family history, the ratio of the number of dominant to recessive inherited pattern was approximately 4:1, which was similar to the result of another study (15).

SPG4 was the most common genotype regardless of inheritance mode and was found in 38.1% (8/21) of patients with a final diagnoses of HSPs, a proportion that was much lower than the 62.0% and 56.4% reported in previous studies (15, 17). Furthermore, the proportion of patients with phenotype of pure form of HSPs among patients diagnosed with SPG4 (5/8, 62.5%) was lower than that reported in another study (18). In this study, approximately 3/4 of patients manifested a phenotype of complex form of HSPs, which might explain the lower frequency of SPG4 detected and the lower proportion of patients with pure form phenotype in patients with SPG4.

In addition to the dominant symptoms of progressive weakness and spasticity of the lower limbs, we found that dysarthria and cerebellar ataxia were the most common symptoms in patients manifesting phenotype of complex form of HSPs with or without definite genetic causes. In those patients with definite diagnoses, signs of dysarthria or cerebellar ataxia were detected in approximately 1/4 of HSPs and 1/2 of SCAs. Gait disturbance was the most common initial symptom in both the patients with HSPs and patients with SCAs. The results were in line with previous studies and reviews showing that the clinical characteristics of SCAs and SPGs were overlapped (7, 10, 11, 15, 16). Pyramidal signs and spasticity could be the first neurological sign before the appearance of cerebellar signs in SCAs and even some cases of SCAs could manifest as the phenotype of pure form of HSPs (10, 12). In this study, there was no causative mutation associated with spastic ataxia (8), such as *SACS/VAMP1/KIF1C/MARS2/MTPAP/AFG3L2(AR)*, detected.

Many subtypes of HSPs have no remarkable changes on brain and spinal cord MRI, while the others have some neuroimaging abnormalities observed on MRI. The “ear of the lynx” sign is a typical MRI characteristic in patients with SPG11 and SPG15 (19). A thin corpus callosum, prominent spinal cord atrophy, ventricular dilatation, white matter hyperintensity, and bilateral T2 hyposignal of the globus pallidus are the other neuroimaging characteristics that can be found in different HSPs subtypes (20). All these neuroimaging findings would be helpful in differentiating HSPs from SCAs, for cerebellar atrophy is the only prominent neuroimaging finding in SCAs (21). However, cerebellar atrophy can also be found in some subtypes of HSPs such as SPG5A (22), SPG7 (20, 23), SPG11 (23, 24), SPG15 (25), SPG20 (26), SPG30 (27), SPG39 (28), and SPG46 (29). Cerebellar atrophy could even be the only abnormal neuroimaging finding in some subtypes of HSPs mentioned above (22, 27, 29). Cerebellar atrophy was observed in one patient with SPG5A in this study.

There is still no consensus genetic diagnostic strategy for HSPs. A practical diagnostic flowchart based on complex or pure forms of HSPs patients with or without a family history, the main clinical and neuroimaging hallmarks, and refereed to specific subtypes of HSPs, followed by the corresponding genetic testing, e.g., Sanger sequencing, gene panel, and MLPA, is the classical gene-by-gene strategy (30). The gene-by-gene strategy used to be the cost-efficient way to diagnose HSPs. There is an increasing awareness that a variety of hereditary diseases have overlapping neurological phenotypes with HSPs. The possibility of causative genetic mutation to be detected is limited by the gene-by-gene strategy. Moreover, due to great advances in NGS technology, the cost of NGS has greatly decreased. In terms of cost and efficiency, the gene-by-gene strategy has no competitive advantage over NGS. Both the targeted gene sequencing and WES are routine genetic diagnostic approaches for HSPs. In particular, WES allows the discovery of new HSP loci to increase the possibility that a causative mutation will be identified, as well as to achieve an earlier diagnosis before the full clinical picture has been manifested, leading to lower expenses on traditional neuroimaging and laboratory testing.

Hereditary spastic paraplegia and SCAs are traditionally designated in separate clinical genetic disease classifications. Due to their overlapping clinical symptoms and neuroimaging findings, it is difficult to differentiate HSPs from SCAs without genetic testing. With the advent of next-generation sequencing, phenotypically unbiased studies have also revealed that HSPs and SCAs not only have overlapping phenotypes, but also share genes (31–33). Additionally, HSP-like phenotypes can also be caused by expansions in triplet-repeat ataxia loci and the abnormal TNRs cannot be detected by targeted gene panel sequencing or by WES in routine (34). The classification system largely frames clinical thinking and genetic workup in clinical practice. Given the common pathophysiological pathways and mechanisms shared by SCAs and HSPs, a proposal to replace the divisive diagnosis-driven classification of SCAs and HSPs has been proposed (35).

In this study, more than one-fifth of cases of suspected HSPs with definite genetic causes were diagnosed as SCAs by the genetic diagnostic strategy of NGS combined with TP PCR. As SCAs were much more frequent than we previously expected, NGS combined with TP PCR might be an efficient selection as the genetic diagnostic strategy for patients clinically suspected of HSPs at present for earlier diagnosis and a higher possibility of causative mutation detected. Unlike targeted gene panels, WES has the strength to detect exon mutations with a hypothesis-free approach compared with the targeted gene panels by allowing for the potential interrogation of many relevant genes. WES combined TP PCR would be an innovative diagnostic algorithms for patients with HSPs-like phenotypes of unknown genetic cause. A large-scale assessment of the diagnostic potential of detecting short tandem repeats expansion from WES data showed that by applying ExpansionHunter with optimized manual curation is capable of detecting some dynamic mutation associated with movement disorders, some genes associated with SCAs were included, such as *ATXN1, ATXN3, ATXN7*, and *NOP56* (36). This suggests that WES might be a better diagnostic tool in patients clinically suspected of HSPs given the possibility to detect repeat expansion compared to target targeted gene panels in clinics. However, the repeat expansion detected by the analysis of the WES data needs to be further validated and the number of repeats should be tested by PCR or TP PCR. A recent study showed that WGS was reliable for detecting repeat expansion with high sensitivity and specificity (13). WGS would be a promising diagnostic tool for the possibility to detecting repeat expansions and deep intronic variants regardless of the high expense.

There were some limitations of this study. This study enrolled a high proportion of patients with sporadic and complex form of HSPs-like phenotype. The percentage of patients with final diagnoses of SCAs might be higher due to the high proportion of patients with complex form of HSPs-like phenotype enrolled in this study. The data from a single center might limit the generalization of the results. The findings of cerebellar atrophy on MRI were interpreted by a senior neuroimaging specialist, but the volume of the cerebellum was not calculated due to the limitations in the neuroimaging data.

## Conclusion

Causative genetic mutations were identified in half of patients with clinically suspected HSPs by NGS and triplet repeat primed PCR. A final diagnosis of HSPs or SCAs was made in 77.7% (21/27) and 22.2% (6/27) of patients with definite causative genetic mutations, respectively. Gait disturbance was the most common initial symptom in both the patients with HSPs and patients with SCAs. The signs of dysarthria and cerebellar ataxia were the most common symptoms in addition to progressive weakness and spasticity of the lower limbs in patients with HSPs. Neuroimaging findings were overlapped between patients with HSPs and patients with SCAs. For the proportion of repeat expansion of genes associated with SCAs detected in patients with definite genetic causes as high as 18.5%, dynamic mutations should be screened in patients with HSPs-like clinical manifestations, especially in patients with the phenotype of complex form of HSPs.

## Data Availability Statement

The datasets presented in this article are not readily available due to ethical restrictions. Requests to access the datasets should be directed to ZZ, ttyyzzq@163.com.

## Ethics Statement

The studies involving human participants were reviewed and approved by Ethics Committees of Beijing Tiantan Hospital. The patients/participants provided their written informed consent to participate in this study.

## Author Contributions

YS and ZZ contributed to the study conception and design. Data collection were performed by YS, AW, BC, SN, and XW. The final diagnoses of cases were made by ZZ and WL. SL interpreted neuroimaging. The first draft of the manuscript was written by YS and the authors commented on previous version of the manuscript. All authors have read and approved the final version of the manuscript.

## Conflict of Interest

The authors declare that the research was conducted in the absence of any commercial or financial relationships that could be construed as a potential conflict of interest.

## Publisher's Note

All claims expressed in this article are solely those of the authors and do not necessarily represent those of their affiliated organizations, or those of the publisher, the editors and the reviewers. Any product that may be evaluated in this article, or claim that may be made by its manufacturer, is not guaranteed or endorsed by the publisher.
