# Supplementary material for: Clinical Features and Genetic Spectrum of Patients With Clinically Suspected Hereditary Progressive Spastic Paraplegia
Source: Front Neurol. 2022 Apr 28;13:872927. doi: 10.3389/fneur.2022.872927 (PMC9097539; doi:10.3389/fneur.2022.872927)
Supplement: Supplementary file 1 [file Data_Sheet_1.PDF]

Supplementary Material

Supplementary Table 1 Gene Panel associated with the phenotype of progressive spastic paraplegia.

KIF5A	RTN2	HSPD1	BSCL2	REEP1	ZFYVE27	ATL1
SLC33A1	SPAST	NIPA1	CPT1C	WASHC5	SPG11	ZFYVE26
ERLIN2	SPG21	B4GALNT1	DDHD1	KIF1A	FA2H	PNPLA6
C19orf12	GJC2	NT5C2	GBA2	AP4B1	AP5Z1	TECPR2
AP4M1	AP4E1	AP4S1	VPS37A	DDHD2	C12orf65	CYP2U1
TFG	CYP7B1	ARL6IP1	AMPD2	ENTPD1	REEP2	IBA57
MAG	CAPN1	FARS2	ATP13A2	UCHL1	SPG7	ALDH18A1
ERLIN1	HACE1	KLC2	CCT5	L1CAM	PLP1	FXN
SNX14	ATXN1	ATXN10	TTBK2	PPP2R2B	KCNC3	PRKCG
ITPR1	TBP	KCND3	ATXN2	TMEM240	PDYN	EEF2
FGF14	AFG3L2	ATXN3	BEAN1	ELOVL4	TGM6	NOP56
ELOVL5	CCDC88C	TRPC3	SPTBN2	CACNA1A	ATXN7	ATXN8
ATXN8OS	CACNA1G	MME	SCYL1	KCNJ10	APTX	TTPA
DNAJC3	FLVCR1	PIK3R5	SAMD9L	ATM	MRE11	PCNA
RNF170	SACS	TDP1	VLDLR	WDR81	CA8	ATP8A2
ATCAY	FRMD4A	KCNA1	CACNB4	SLC1A3	SIL1	CAMTA1
ABHD12	VAMP1	KIF1C	MARS2	MTPAP	VWA3B	TDP2
UBA5	ANO10	SYT14	GRM1	RUBCN	STUB1	CWF19L1
GRID2	ZNF592	TPP1	ABCB7	ATP2B3	MTTP	COQ8A
RETREG1	PRKN	FLNA	CHMP1B	ATN1	ATP1A3	DNMT1
GFAP	IFRD1	ITM2B	NOL3	PAX6	POLG	PTEN
SCN1A	SLC2A1	TUBB4A	VHL	SETX	SYNE1	WWOX
COL18A1	GOSR2	PRICKLE1	SCARB2	KCTD7	PEX10	EXOSC3
DNAJC19	ERCC8	ERCC6	PLA2G6	DARS2	CLCN2	SLC17A5
POLR3A	PRPS1	SLC9A6	MECP2	FMR1	SPG20	SLC16A2
PGAP1	RAB3GAP2	MARS	BICD2	LYST	ETFDH	ABCD1
TH	GCH1	MUT	MMACHC	GCDH	MTHFR	ARG1
ARSA	CBS	IDS	PCCA	PCCB	ETHE1	GAD1
ARX	ATRX	AAAS	CYP27A1	SLC25A15	GALC	IDUA
DARS	GJA1	SLC30A10	NEU1	ALDH3A2	ALG6	ALS2
ATP7B	CSTB	EIF2B2	EIF2B3	EIF2B5	FOXC1	HSD17B4
PMM2	RNF216	SEPSECS	TSEN2	TSEN34	TSEN54	VPS53
CASK	CUL4B	DKC1	INPP5E	OPHN1	PRNP	SOD1
HEXA	KANK1	IFIH1	TENM3	PHYH	LMNB1	NBN
ATP6AP2	USP8	WDR48	ARSI	LCOR	FLRT1	ZFR

EMC1	APP	NOTCH3	HTRA1	ADD3	CTC1	TUBB3
KIF5C	KIF2A	TUBG1	TUBB	CST3	GSN	BCAP31
MFF	RANBP2	TBCD	SERPINI1	SLC25A12	JAM3	HYLS1
ACO2	DNM1L	FOLR1	PANK2	FTL	WDR45	COASY
RERE	MPDZ	ARFGEF2	ERMARD	POU1F1	ZIC3	

Supplementary Table 2 The primer sequences of all know SCAs genes are designed using reference sequences from GenBank and they are listed below.

Subtype	Gene	Primer
**SCA1**	ATXN1	5'-GGGCCACCACTCCATCCCAG-3' (sense)
		5'-GCGGAGAACTGGAAATGTGGAC-3'(antisense)
**SCA2**	ATXN2	5'-GGGCCCCTCACCATGTCG-3' (sense)
		5'-CGGGCTTGCGGACATTGG-3' (antisense)
**SCA3**	ATXN3	5'-CCAGTGACTACTTTGATTCG-3' (sense)
		5'-GTAACCTTGCTCCTTAATCC-3' (antisense)
**SCA6**	CACNA1A	5'-CACGTGTCCTATTCCCCTGTGATCC-3' (sense)
		5'-TGGGTACCTCCGAGGGCCGCTGGTG-3' (antisense)
**SCA7**	ATXN7	5'-TAGGAGCGGAAAGAATGTCG-3' (sense)
		5'-AGCCTCAACCCACAGATTCC-3' (antisense)
**SCA8**	ATXN8	5'-TTTGAGAAAGGCTTGTGAGG ACTGAGAATG-3'(sense)
		5'-GGTCCTTCATGTTAGAAAACCTGGCT-3' (antisense)
**SCA10**	ATXN10	5'-CTCCAGTGCAACCACTTTTAGA-3' (sense)
		5'-AGGCAGGAGAATTGCTTGAA-3' (antisense)
**SCA12**	PPP2R2B	5'-TGCTGGGAAAGAGTCGTG-3' (sense)
		5'-GCCAGCGCACTCACCCTC-3' (antisense)
**SCA17**	TBP	5'-GACCCCACAGCCTATTCAGA-3' (sense)
		5'-TTGACTGCTGAACGGCTGCA-3' (antisense)
**SCA36**	NOP56	5'- TTTCGGCCTGCGTTCGGG -3' (sense)
		5'- AACGCAACCTCAGCGTCT-3' (antisense)
**DRPLA**	ATN1	5'-CACCAGTCTCAACACATCACCATC-3' (sense)
		5'-CCTCCAGTGGGTGGGGAAATGCTC-3' (antisense)
**FRDA**	FXN	5'-GGCTTAAACTTCCCACACGTGTT-3' (sense)
		5'-AGGACCATCATGGCCACACTT-3' (antisense)

Subtype	TP-F Sequence	TP-R Sequence	TP-U Sequence	Label
**SCA8**	CTGGGTCCTTCATGTTAGAAAACCT	TACGCATCCCAGTTTGAGACGCAGCAGCAGCAGCAG	TACGCATCCCAGTTTGAGACG	TP-F FAM
**SCA10**	CAGATGGCAGAATGATAAACTCAA	TACGCATCCCAGTTTGAGACGAGAATAGAATAGAATAGAATAGAAT	TACGCATCCCAGTTTGAGACG	TP-F FAM
**SCA36**	TTTCGGCCTGCGTTCGGG	TACGCATCCCAGTTTGAGACGCAGGCCCAGGCCCAGGCCCAGGCC	TACGCATCCCAGTTTGAGACG	TP-F FAM
**FRDA**	GGGATTGGTTGCCAGTGCTTAAAAGTTAG	TACGCATCCCAGTTTGAGACGTTCTTCTTCTTCTTCTTCTTC	TACGCATCCCAGTTTGAGACG	TP-F FAM

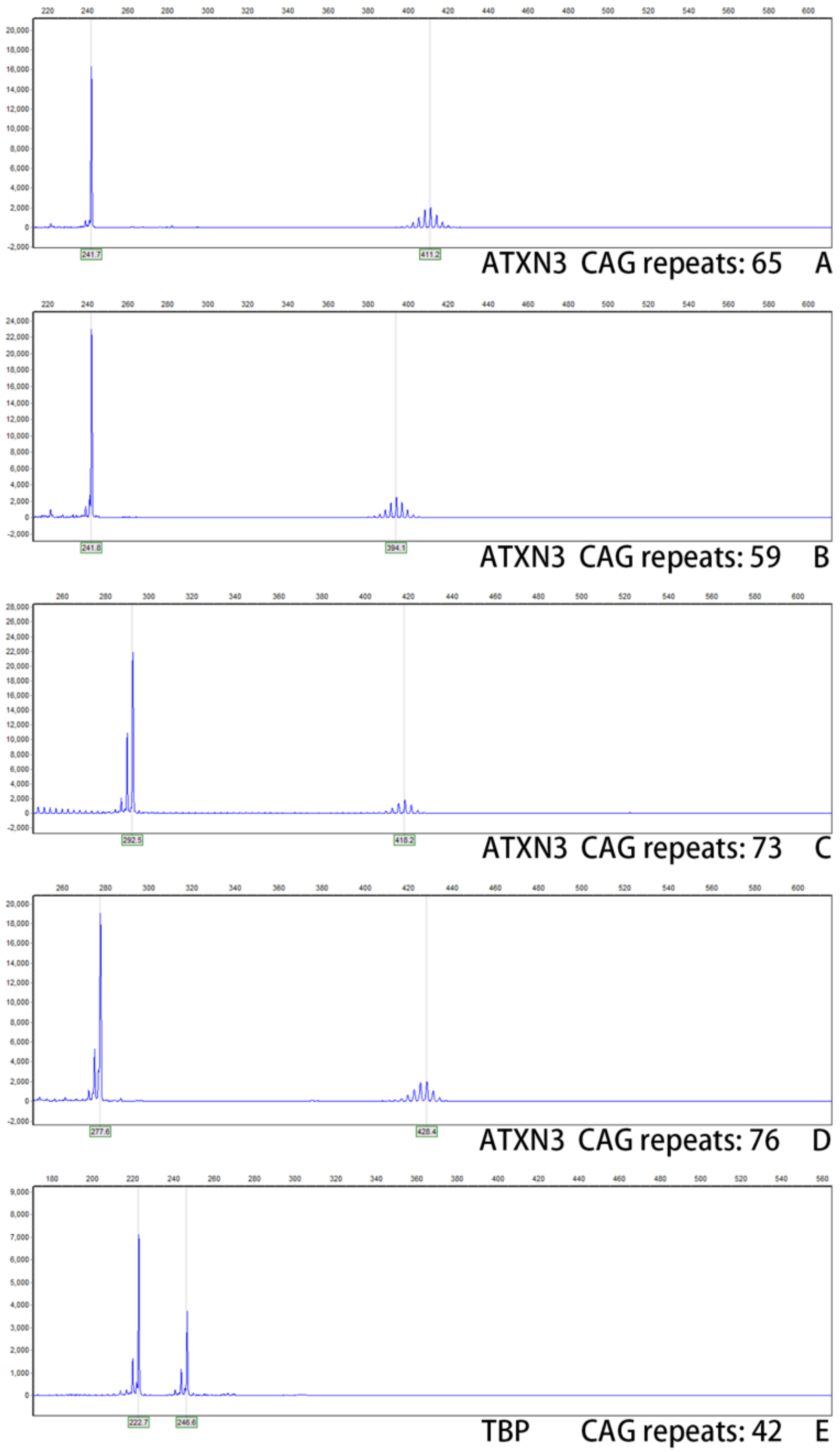

Supplementary Figure 1 Abnormal CAG repeat expansion detected by TP PCR in patients with SCA3 (A, B, C, D) and SCA17 (E).
